# Development and validation of MRI-based radiomics signatures as new markers for preoperative assessment of EGFR mutation and subtypes from bone metastases

**DOI:** 10.1186/s12885-022-09985-4

**Published:** 2022-08-13

**Authors:** Ying Fan, Yue Dong, Xinyan Sun, Huan Wang, Peng Zhao, Hongbo Wang, Xiran Jiang

**Affiliations:** 1 School of Intelligent Medicine, China Medical University, Shenyang, Liaoning 110122 People’s Republic of China; 2 Department of Radiology, Cancer Hospital of China Medical University, Liaoning Cancer Hospital and Institute, Shenyang, Liaoning 110042 People’s Republic of China; 3 Radiation Oncology Department of Thoracic Cancer, Liaoning Cancer Hospital and Institute, Shenyang, Liaoning 110042 People’s Republic of China; 4 Department of Radiology, Shengjing Hospital of China Medical University, Shenyang, 110004 People’s Republic of China

**Keywords:** EGFR, Spinal metastasis, CET1 MRI, Lung adenocarcinoma

## Abstract

**Background:**

This study aimed to develop and externally validate contrast-enhanced (CE) T1-weighted MRI-based radiomics for the identification of epidermal growth factor receptor (EGFR) mutation, exon-19 deletion and exon-21 L858R mutation from MR imaging of spinal bone metastasis from primary lung adenocarcinoma.

**Methods:**

A total of 159 patients from our hospital between January 2017 and September 2021 formed a primary set, and 24 patients from another center between January 2017 and October 2021 formed an independent validation set. Radiomics features were extracted from the CET1 MRI using the Pyradiomics method. The least absolute shrinkage and selection operator (LASSO) regression was applied for selecting the most predictive features. Radiomics signatures (RSs) were developed based on the primary training set to predict EGFR mutations and differentiate between exon-19 deletion and exon-21 L858R. The RSs were validated on the internal and external validation sets using the Receiver Operating Characteristic (ROC) curve analysis.

**Results:**

Eight, three, and five most predictive features were selected to build RS-EGFR, RS-19, and RS-21 for predicting EGFR mutation, exon-19 deletion and exon-21 L858R, respectively. The RSs generated favorable prediction efficacies for the primary (AUCs, RS-EGFR vs. RS-19 vs. RS-21, 0.851 vs. 0.816 vs. 0.814) and external validation (AUCs, RS-EGFR vs. RS-19 vs. RS-21, 0.807 vs. 0.742 vs. 0.792) sets.

**Conclusions:**

Radiomics features from the CE MRI could be used to detect the EGFR mutation, increasing the certainty of identifying exon-19 deletion and exon-21 L858R mutations based on spinal metastasis MR imaging.

**Supplementary Information:**

The online version contains supplementary material available at 10.1186/s12885-022-09985-4.

## Introduction

Lung cancer is the most frequently diagnosed cancer worldwide and continues to increase in both incidence and mortality [1–3]. Non-small-cell lung cancer (NSCLC) represents approximately 85% of all lung cancer cases, of which lung adenocarcinoma (LUAD) is the most common histologic subtype [4]. Identification of epidermal growth factor receptor (EGFR) mutations has important therapeutic implications in LUAD [5] because tyrosine kinase inhibitors (TKIs) have been effective for LUAD with EGFR mutations [6]. Patients with EGFR mutations are more sensitive to EGFR-TKIs than those with EGFR wild-type [7]. EGFR mutations mostly occur in exons 18, 19, 20, and 21, among which 19 deletions and 21 L858R were the most common subtypes [8, 9] and accounted for approximately 90% of EGFR mutation cases [10]. Patients who carry the EGFR mutation in exon 19 or 21 often have a higher radiographic response rate to EGFR-TKIs [11], and higher response rate to afatinib, erlotinib, and gefitinib [11], resulting in longer survival time [12, 13]. Therefore, early detection of EGFR mutations and subtypes is of great significance in therapeutic decisions.

Bone metastasis often occurs in LUAD, and the prevalence rate is approximately 30–40% [14]. In clinical practice, if tissue biopsies of primary LUAD are impossible to perform, a spinal metastatic lesion can be an important alternative for assessing EGFR mutation status [15]. However, biopsy of spinal metastases may damage the nerve fibers in the spinal cord and increases the risk of metastases [16, 17]. Magnetic resonance imaging (MRI) is a noninvasive method that allows direct visualization of bone marrow abnormalities [18]. Contrast-enhanced (CE) MRI can clearly display enhanced regions within the tumor, distinguishing necrosis from solid tumors [19]. A previous study showed that CE MRI is sensitive for the diagnosis of small lesions of bone metastases [20]. Although MRI is a powerful diagnostic technique for NSCLC [21], a biomarker has not been created to detect the EGFR mutation in the spinal metastasis by visual examination of the MRI image.

Radiomics refers to the quantitative analysis of medical imaging, with the capability of obtaining valuable information from imaging data that can be applied within clinical decision support systems for developing diagnostic and predictive models [22, 23]. Many radiomic approaches have been used to detect EGFR mutations in NSCLC. However, the majority of previous studies have focused on EGFR mutations in primary lung cancer [24–28]. Recent efforts have evaluated the associations between radiomics features derived from metastatic lesions in the brain and EGFR mutation status [29–32]. Recent reports have revealed that MRI features of bone metastasis are also related to EGFR mutation status [33, 34]. However, these studies only assessed non-CE MR data based on limited sample sizes and lack of external samples to verify their findings, which is inherently limiting. To the best of our knowledge, the relationship between CE MRI of bone metastasis and EGFR mutation status has not yet been clarified. Therefore, this study aimed to explore the value of CE MRI-based radiomics in an attempt to identify EGFR mutations and subtypes based on spinal metastasis.

## Methods

### Patients

**Fig. 1:**
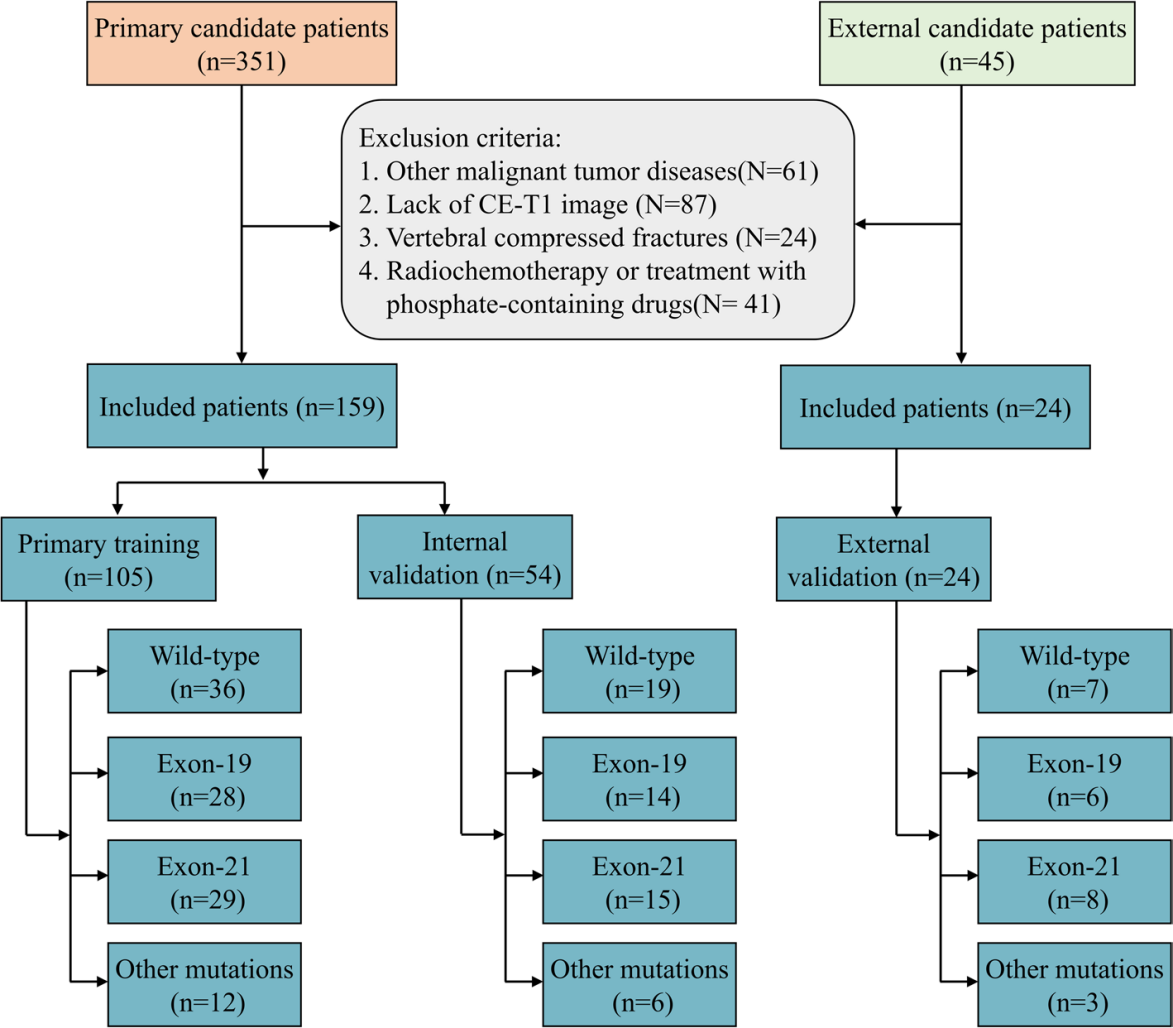
Illustration of the patients’ recruitment in this study

This retrospective study was approved by the Medical Ethics Committee of Liaoning Cancer Hospital and Institute, and the requirement for informed consent was waived. A primary set of 159 patients was enrolled from Liaoning Cancer Hospital and Institute between Jan. 2017 and Sep.2021. An external set was established with 24 patients from Shengjing Hospital between Jan. 2017 and Sep.2021. All patients were pathologically diagnosed with spinal metastases from primary lung adenocarcinoma. The inclusion criteria were as follows: (i) age > 18; (ii) CET1 MRI scans before surgery; and (iii) complete clinical data. The exclusion criteria were as follows: (i) other malignant tumor diseases; (ii) radiochemotherapy or treatment with phosphate-containing drugs; and (iii) vertebral compressed fractures. The primary set was divided into a training set and an internal validation set at a 2:1 ratio by stratified sampling. The external set was used for independent validation. Clinical data for patients including age, sex, smoking status, performance status, carcinoembryonic antigen (CEA), cytokeratin (CYFRA), and neuron-specific enolase (NSE) were collected from the hospital’s medical system. Figure 1 shows the patient recruitment process.

### MRI acquisition and metastasis delineation

MRI scans in primary and external cohorts were performed using the 3.0 T scanner (Siemens Magnetom Trio, Erlangen, Germany). The contrast-enhanced T1-weighted MRI parameters were as follows: echo time (TE) = 9.0 ms, repetition time (TR) = 550 ms, slice thickness = 4 mm, scan interval = 4.4 mm, field of view = 640 × 640 mm and matrix size = 256 × 256. The contrast agent (Gd-DTPA-MBA, Omniscan, GE Healthcare) was injected intravenously at a dose of 0.2 mmol/kg. Subsequently, 20 mL of saline was flushed at a rate of 2.0 mL/s.

Spinal metastases were manually segmented by a radiologist (Xinyan Sun) with three years of experience to generate regions of interest (ROIs) along the border of the metastatic lesions in the CET1 MR image. The segmentations were crosschecked by a senior radiologist (Yue Dong) with 17 years of experience. The delineated ROIs were stored in a NII format.

### Feature extraction

The delineated ROIs on the MRI slices were used to extract radiomics features using the pyradiomics package in Python version 3.6. A set of 1967 features were extracted, which consisted of first-order statistical, shape-based and textural feature families. Various filters including wavelet, square, squareroot, gradient, exponential, logarithmic, local binary pattern, and Laplacian of Gaussian were used to transform the original MR images. Then, first-order statistical and textural features were extracted from the transformed images to obtain the filtered features. Detailed protocols can be found in a previous study [35] and the pyradiomics document, which is available at https://pyradiomics.readthedocs.io/en/latest/.

### Feature selection and radiomics model construction

To estimate stability and reproducibility of all extracted features, intraclass correlation coefficient (ICC) analysis was performed to access interobserver agreements of the features [36]. Thirty patients were randomly invited to perform ICC analysis, 15 with EGFR wild-type and 15 with EGFR mutation. The cut-off value was set as 0.85, and features with ICC > 0.85 were retained. Subsequently, these candidate features were further evaluated using the Mann–Whitey *U* test. Features with *P-value* < 0.05 were retained, then further selected by least absolute shrinkage and selection operator (LASSO) regression. The optimal lambda was selected with 5-fold cross-validation. By integrating the selected imaging features with the corresponding non-zero LASSO coefficient, radiomics signatures (RSs) were developed to predict EGFR mutations and subtypes (RS-EGFR, RS-19, and RS-21) by logistic regression using the “glmnet” package in R version 3.6. All models were constructed on the primary training set and tested on both the internal and external validation sets.

### Statistical analysis

**Fig. 2:**
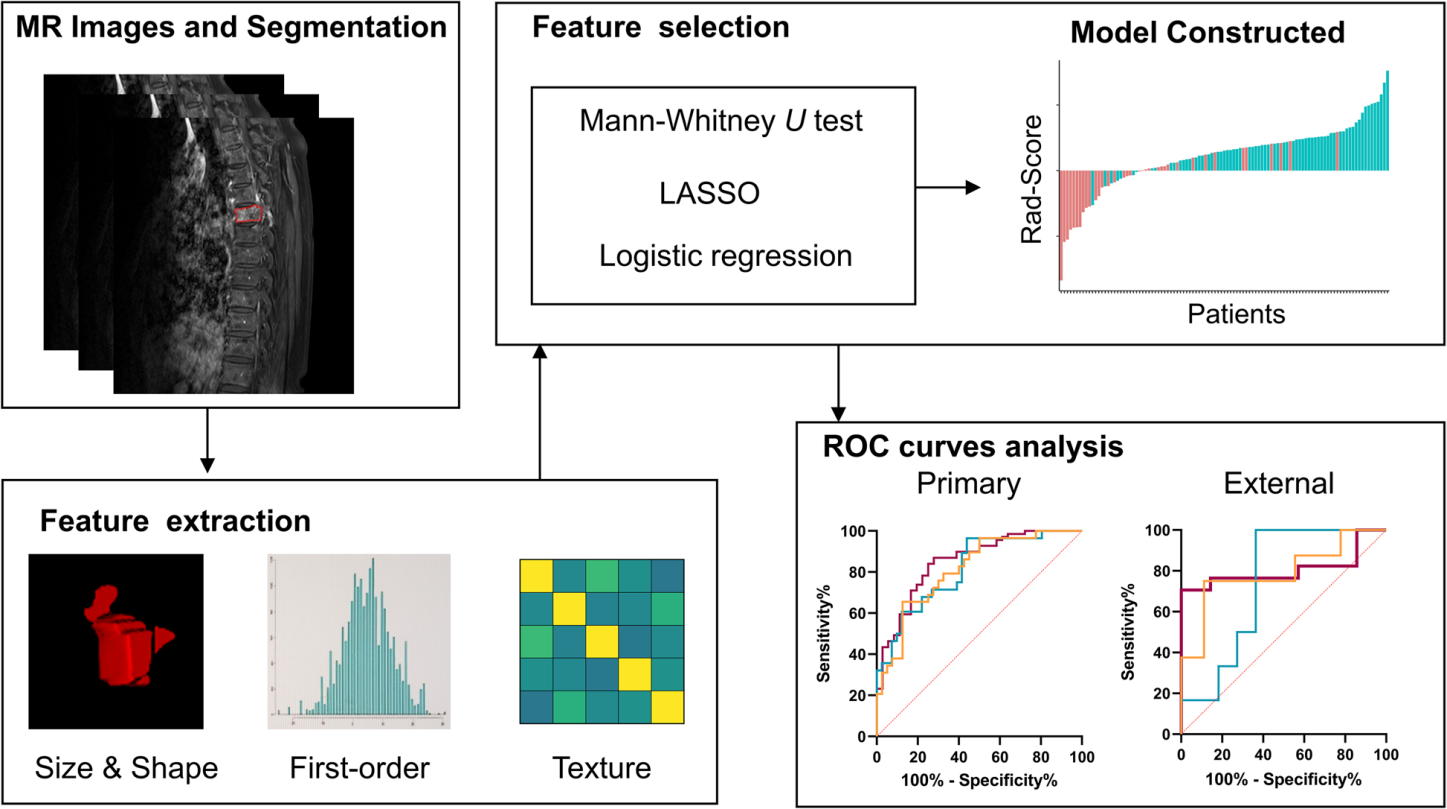
Workflow of this study

All statistical analyses were performed using R and MedCalc 20.0.14 (MedCalc Inc.). A two-sided *P*-value < 0.05 was considered significant. Statistical differences in distributions between patients and variables were evaluated using the *t*-test, Mann–Whitney U test, and chi-square test, as appropriate. The Youden index [37] was used to determine the optimal cutoff values in the ROC analysis. The area under the ROC curve (AUC), accuracy, specificity (true negative rate), and sensitivity (true positive rate) were calculated to assess the prediction capabilities. Figure 2 shows the workflow of this study.

## Results

### Patients’ characteristics

The clinical characteristics of the patients are summarized in Table 1. We included 62 patients (33.9%) with wild-type EGFR and 121 patients (66.1%) with EGFR mutations. Among the EGFR mutant patients, 48 patients (39.7%) had EGFR mutations in exon 19, 52 patients (43.0%) had EGFR mutations in exon 21, and 21 patients (17.3%) had EGFR mutations in exon 18/20. There were no significant differences (*P* > 0.05) in terms of age, sex, smoking status, PS, CEA, CYFRA, and NSE levels between the EGFR wild-type and EGFR-mutant groups in the primary and external sets.

### Radiomics feature selection

**Fig. 3:**
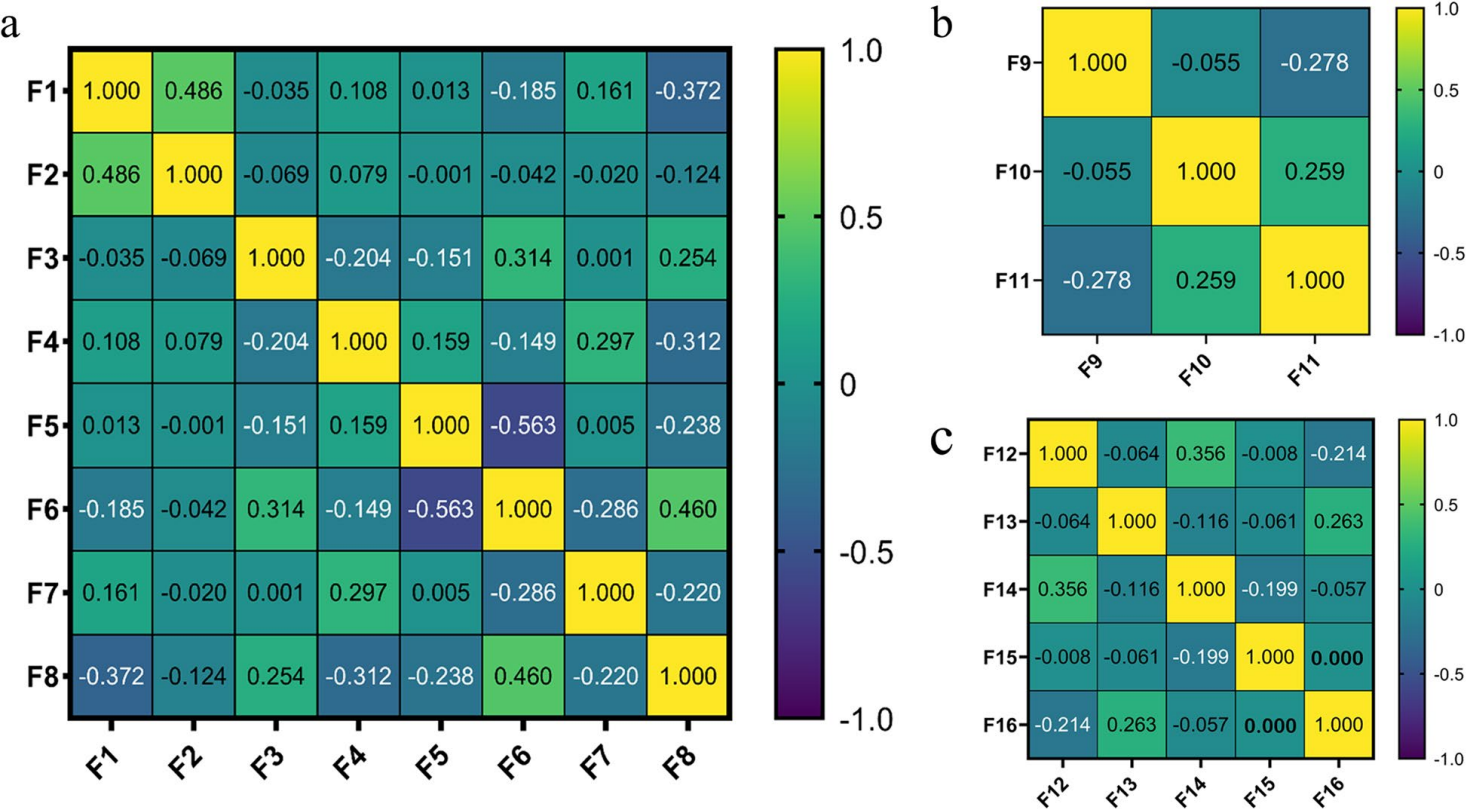
Pearson correlation coefficient matrix of the selected features (F1-F16) for the prediction of RS-EGFR (**a**), RS-19 (**b**) and RS-21 (**c**)

To predict EGFR mutations, we selected eight features as the most important predictors. All features belonged to the textural feature family and showed good prediction performance in terms of AUCs. The features showed statistically significant differences (*P* < 0.05) in predicting EGFR mutations. To predict exon-19 deletion and exon-21 L858R mutations, three and five most important features were selected, respectively. All features generated an acceptable predictive performance, with AUCs ranging from 0.621 to 0.723. Table 2 lists the prediction performance of the selected features. Figure 3 shows the correlations among the selected features.

**Table 2:** Prediction performance of the most important features for predicting the EGFR mutation, exon-19 deletion and exon-21 L858R

Feature	Mean ± SD				AUC	***P***
	EGFR mutant	EGFR wild-type	Exon-19 deletion	Exon-21 L858R		
exponential_glszm_SmallArea Emphasis (F1)	−0.20 ± 0.87	0.38 ± 1.13	–	–	0.652	0.010
exponential_ngtdm_Strength (F2)	−0.19 ± 0.55	0.36 ± 1.47	–	–	0.629	0.029
log-sigma-3-0-mm-3D_glcm_InverseVariance (F3)	0.17 ± 1.00	−0.32 ± 0.92	–	–	0.649	0.009
log-sigma-5-0-mm-3D_glrlm_Long RunHighGrayLevelEmphasis (F4)	−0.15 ± 0.92	0.29 ± 1.1	–	–	0.640	0.019
wavelet-HHL_glcm_ ClusterShade (F5)	0.11 ± 1.09	−0.2 ± 0.77	–	–	0.632	0.017
wavelet-HHL_glrlm_GrayLevel NonUniformityNormalized (F6)	0.10 ± 1.15	−0.19 ± 0.59	–	–	0.633	0.032
wavelet-HHL_glrlm_Long RunHighGrayLevelEmphasis (F7)	−0.18 ± 0.64	0.34 ± 1.41	–	–	0.624	0.048
wavelet-LLL_glszm_Small AreaLowGrayLevelEmphasis (F8)	−0.03 ± 0.68	0.07 ± 1.44	–	–	0.658	0.011
original_shape_Elongation (F9)	–	–	0.42 ± 0.85	–	0.723	< 0.001
square_glrlm_LongRunHighGray LevelEmphasis (F10)	–	–	0.45 ± 1.40	–	0.675	0.011
wavelet_LLL_firstorder_Skewness (F11)	–	–	0.38 ± 1.27	–	0.647	0.056
lbp-3D-k_glszm_ SmallAreaLowGrayLevelEmphasis (F12)	–	–	–	0.23 ± 0.89	0.628	0.067
log-sigma-3-0-mm-3D_glszm_ SmallAreaLowGrayLevelEmphasis (F13)	–	–	–	0.29 ± 1.34	0.673	0.008
log-sigma-5-0-mm-3D_glcm_Imc2 (F14)	–	–	–	0.33 ± 0.65	0.645	0.031
square_glszm_SizeZoneNonUniformityNormalized (F15)	–	–	–	0.29 ± 0.77	0.621	0.078
wavelet-LHL_firstorder_Median (F16)	–	–	–	0.39 ± 0.66	0.639	0.038

**Table 1:** Clinical characteristics of the patients in the primary and external validation sets

	Primary training (***n =*** 105)			Internal validation (***n =*** 54)			External validation (***n =*** 24)		
Characteristic	EGFR mutant (***n =*** 69)	EGFR wild-type (***n =*** 36)	***p***	EGFR mutant (***n =*** 35)	EGFR wild-type (***n =*** 19)	***p***	EGFR mutant (***n =*** 17)	EGFR wild-type (***n =*** 7)	***p***
Age (Mean ± SD)	58.71 ± 9.34	57.14 ± 11.28	0.448	58.14 ± 12.11	59.21 ± 8.95	0.738	59.06 ± 7.78	60.14 ± 5.61	0.742
Sex			0.080			0.799			0.202
Male	26 (37.7%)	20 (55.6%)		19 (54.3%)	11 (57.9%)		5 (29.4%)	4 (57.1%)	
Female	43 (62.3%)	16 (44.4)		16 (45.7%)	8 (42.1%)		12 (70.6%)	3 (42.9%)	
Smoking status			0.063			0.532			0.344
Yes	20 (29.0%)	17 (47.25)		10 (28.6%)	7 (36.8%)		4 (23.5%)	3 (42.9%)	
No	49 (71.0%)	19 (52.8%)		25 (71.4%)	12 (63.2%)		13 (76.5%)	4 (57.1%)	
PS Score			0.553			0.645			0.551
0	8 (11.6%)	2 (5.6%)		3 (8.5%)	1 (5.3%)		3 (17.6%)	3 (42.9%)	
1	48 (69.6%)	29 (80.6%)		22 (62.9%)	15 (78.9%)		11 (64.7%)	3 (42.9%)	
2	10 (14.5%)	3 (8.3%)		8 (22.9%)	2 (10.5%)		2 (11.8%)	1 (14.2%)	
3	3 (4.3%)	2 (5.6%)		2 (5.7%)	1 (5.3%)		1 (5.9%)	0 (0.0%)	
CEA (Mean ± SD)	127.01 ± 225.86	118.40 ± 184.17	0.364	84.4 ± 178.92	107.57 ± 276.57	0.738	102 ± 132.94	81.22 ± 124.14	0.187
CYFRA (Mean ± SD)	8.66 ± 13.51	10.51 ± 10.12	0.473	9.8 ± 13.09	12.19 ± 10.34	0.140	14.18 ± 19.25	6.61 ± 3.19	0.852
NSE (Mean ± SD)	21.01 ± 14.59	21.47 ± 11.03	0.787	21.44 ± 16.73	15.22 ± 6.71	0.258	28.11 ± 28.34	18.15 ± 5.99	0.710

*SD* Standard deviation, PS Performance status, *CEA* Carcinoembryonic antigen, *CYFRA* Cytokeratin, *NSE* Neuron-specific enolase

**Table 3:** Prediction performance of RS-EGFR, RS-19 and RS-21

RS	Primary training				Internal validation				External validation			
	AUC (95%)	ACC	SEN	SPE	AUC (95%)	ACC	SEN	SPE	AUC (95%)	ACC	SEN	SPE
RS-EGFR	0.851 (0.774-0.921)	0.800	0.722	0.870	0.780 (0.645-0.916)	0.741	0.771	0.737	0.807 (0.595-0.938)	0.625	0.765	0.857
RS-19	0.816 (0.716-0.917)	0.739	0.964	0.561	0.789 (0.636-0.942)	0.600	0.786	0.714	0.742 (0.478-0.919)	0.623	0.833	0.636
RS-21	0.814 (0.714-0.914)	0.739	0.655	0.875	0.770 (0.609-0.931)	0.657	0.800	0.700	0.792 (0.530-0.946)	0.529	0.750	0.889

*AUC* Area under the receiver operating characteristic curve, *ACC* Accuracy, *SEN* Sensitivity, *SPE* Specificity, *SD* standard deviation

### Radiomics signature construction and validation

**Fig. 4:**
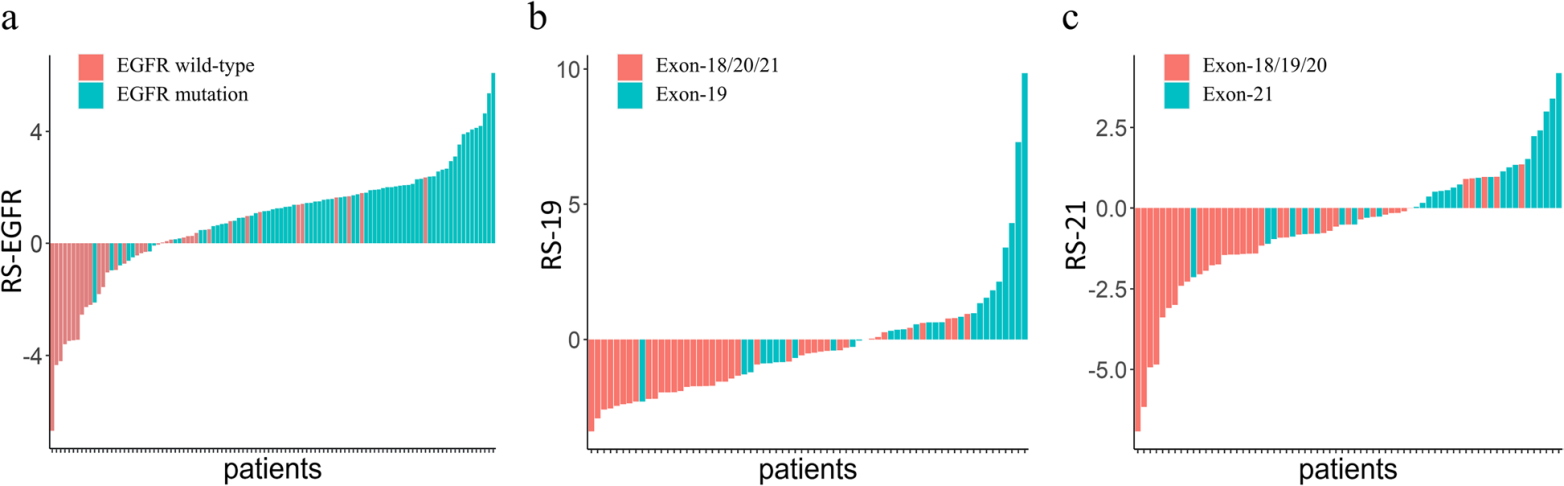
Developed RSs for the prediction of EGFR mutation (**a**), exon-19 deletion (**b**) and exon-21 L858R (**c**) mutation

We used the most important selected features to incorporate their nonzero coefficients to build radiomic signatures (RSs). The developed RS-EGFR aims to distinguishing EGFR mutant patients from EGFR wild-type patients. The developed RS-EGFR aims to distinguish patients with EGFR mutations from patients with wild-type EGFR. The developed RS-19 aims to distinguish patients with EGFR mutations in exon 19 from those with EGFR mutations in exon 18/20/21. RS-21 aims to distinguish patients with EGFR mutations in exon 21 from those with EGFR mutations in exons 18/19/20. Figure 4 indicates that our RSs can effectively differentiate between patients with wild-type EGFR, EGFR mutations, exon-19 deletion and exon-21 L858R mutation.

### Radiomics model performance

**Fig. 5:**
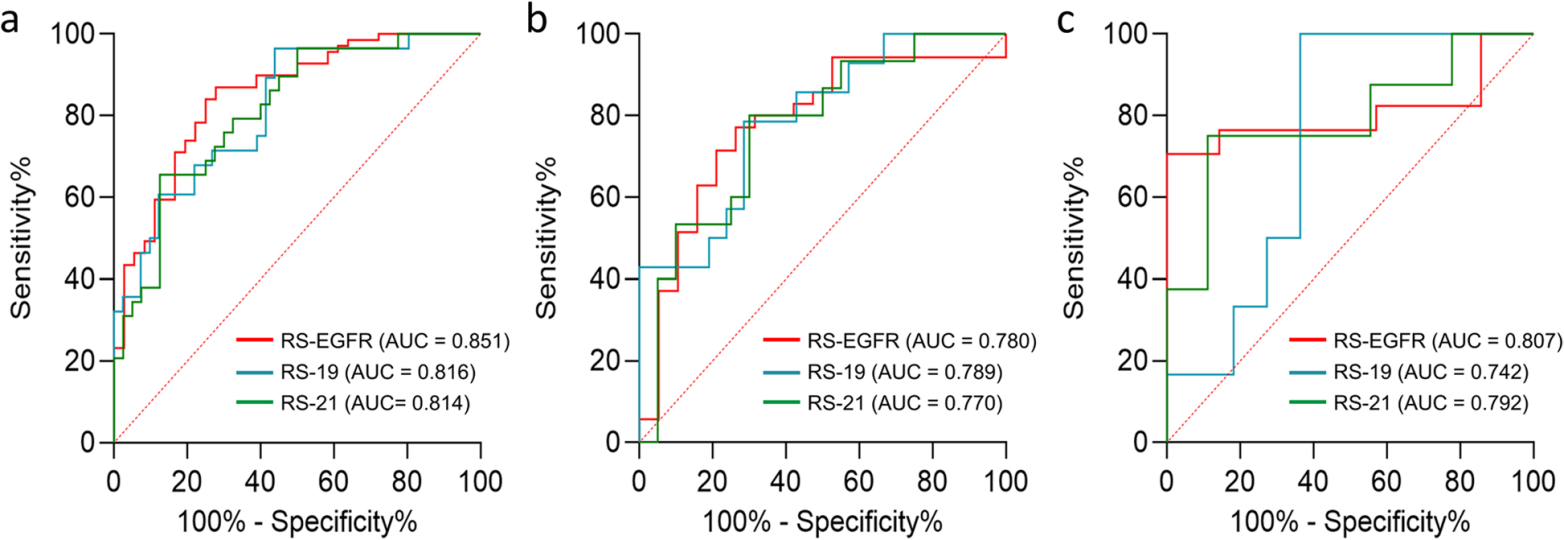
ROC curves of the developed RS- EGFR, RS-19 and RS-21 in the primary training (**a**), internal validation (**b**) and external validation (**c**) sets

Table 3 presents the prediction performance of the RS-EGFR, RS-19, and RS-21 in the primary and external validation sets. ROC curves of the RSs were shown in Fig. 5. For the prediction of EGFR mutation, the RS-EGFR generated AUCs of 0.851 and 0.807 in the primary and external validation sets, respectively. To predict the exon-19 deletion and exon-21 L858R mutations, the RS-19 and RS-21 yielded predictive AUCs of 0.816 and 0.742 and 0.821 and 0.764 in the primary and external validation sets, respectively.

## Discussion

Noninvasive evaluation of EGFR mutation status and subtypes based on bone metastasis is of great clinical significance, yet it has not been well studied. Previous studies related to our study mainly focused on primary lesions of lung adenocarcinoma and highlighted that radiomics can be helpful in predicting EGFR mutations and subtypes mainly from medical imaging [10, 21, 26, 38]. Recently, CE MRI has been shown to provide information about the vascularity of the tissue and its surrounding environment [39]. A previous report indicated that CE MRI can reflect an increase in blood vessels of the tumor in the paraspinal muscles and spinal canal, thereby assessing the spread of the tumor outside the skeletal system [40]. To the best of our knowledge, this study is the first attempt to investigate CE MRI-based radiomics for the assessment of EGFR mutation status in bone metastases.

Eight features were identified as the most predictive for EGFR mutations, all of which were textural features. This may suggest that the intratumoral heterogeneity within the spinal metastasis was related to the EGFR mutation status, considering that textural features can reflect intratumoral non-uniformity [41]. This was partly consistent with Lindberg’s study, which demonstrated the relationship between EGFR mutations and heterogeneity and aggressiveness of lung cancer [42]. To predict exon-19 deletion and exon-21 L858R, we identified three and five most important features from the CET1 MRI, respectively. Notably, most of the selected features (four of five) for predicting exon-21 L858R belong to the textural feature class. This may indicate that heterogeneity distributions within the metastasis were different between tumor carrying exon-19 deletion and exon-21 L858R mutations. The selected original_shape_elongation feature describes the ROI shape. A higher value of this feature indicates a rounder tumor shape. Our findings suggest that tumors with exon-19 deletion tend to be rounder compared with that carrying exon-21 L858R or EGFR wild-type.

Before this study, Jiang et al. reported that non-enhanced MRI-based radiomics can be used to assess the EGFR mutation status in bone metastases but failed to analyze the specific mutation sites [33]. Previous investigations on the assessment of exon-19 deletion and exon-21 L858R are limited. Li et al. [10] generated an AUC of 0.79 for detecting exon-19 deletion and exon-21 L858R using radiomics based on primary lung adenocarcinoma. Cao et al. [43] yielded an AUC of 0.901 for detecting exon-19 deletion and exon-21 L858R based on metastasis from the original lung adenocarcinoma. However, the small sample size, single-center data and lack of external validation all results in low clinical values of their findings. This study first predicted the EGFR mutation and then further assessed whether the mutation was located in exon 19 or 21. Our results revealed that features from CE MRI have good potential to detect EGFR mutations and subtypes.

Furthermore, we sought to explore the association between clinical factors and EGFR mutation status, and found that no clinical factor was associated with the EGFR mutation status and mutation subtypes in both primary and external cohorts. This was inconsistent with previous reports that indicated that age and smoking were highly correlated with EGFR mutations [26, 40, 44] and subtypes [10]. CEA level and sex were also previously suggested as independent predictors of EGFR mutation status [25, 45]. However, there were some studies supported our findings. Ren et al. [34], Kim et al. [39] and Zhang et al. [44] also found that age, smoking, CEA level and sex were not correlated with the EGFR mutation status in their datasets.

### Limitation

This study had some limitations. First, although the findings were externally validated on an independent set, the sample size was small because of data collection challenges. The most important features identified on CET1 MRI will need to be verified on a large scale. Second, some important MRI sequences (e.g., T1-weighted, T2-weighted, and T2-weighted fat-suppressed MRI) were not included. Lastly, this study only evaluated the EGFR mutation status before treatment. Assessment of resistant EGFR-T790M mutations in *EGFR* gene is also important in clinical practice to improve individual treatment management.

## Conclusion

In conclusion, this study assessed CET1 MRI-based radiomics for predicting EGFR mutation and subtypes based on the bone metastasis. The developed models performed well on the external set, which may indicate good potential in future clinical applications.

## Supplementary Information

**Additional file 1.**

## Data Availability

The datasets generated and/or analysed during the current study are not publicly available due to restrictions but are available from the corresponding author on reasonable request.

## Abbreviations

AUC: Area under the curve
ROC: Receiver operating characteristic
EGFR-TKIs: Epidermal growth factor receptor-tyrosine kinase inhibitors
NSCLC: Non-small-cell lung carcinoma
MRI: Magnetic resonance imaging
LASSO: Least absolute shrinkage and selection operator
AIC: Akaike information criterion
EGFR: Epidermal growth factor receptor
CET1: Contrast-enhanced T1-weighted imaging
ICC: Intraclass correlation coefficient (ICC)
PS: Performance status
CEA: Carcinoembryonic antigen
CYFRA: Cytokeratin
NSE: Neuron-specific enolase
LUAD: Lung adenocarcinoma
