# Supplementary material for: Development and validation of MRI-based radiomics signatures as new markers for preoperative assessment of EGFR mutation and subtypes from bone metastases
Source: BMC Cancer. 2022 Aug 13;22:889. doi: 10.1186/s12885-022-09985-4 (PMC9375915; doi:10.1186/s12885-022-09985-4)
Supplement: Supplementary file 1 — Additional file 1. [file 12885_2022_9985_MOESM1_ESM.docx]

**The Formulas of the developed radiomics models**

RS-EGFR = -3078.743-4.873929×exponential_glszm_SmallAreaEmphasis-0.9978840× exponential_ngtdm_Strength+14.92238×log-sigma-3-0-mm-3D_glcm_InverseVariance-0.001164395×log-sigma-5-0-mm-3D_glrlm_LongRunHighGrayLevelEmphasis-117.6612×wavelet-HHL_glcm_ClusterShade-6157.136×wavelet-HHL_glrlm_GrayLevel NonUniformityNormalized-0.1235524×wavelet-HHL_glrlm_Long RunHighGrayLevelEmphasis- 376.3667×wavelet-LLL_glszm_Small AreaLowGrayLevelEmphasis

RS-19 = -1.56251364-2.46002950×original_shape_Elongation+0.03045322 ×square_glrlm_LongRunHighGrayLevelEmphasis+1.61180958×wavelet_LLL_firstorder_Skewness

RS-21 = -0.5619225+0.9021561×lbp-3D-k_glszm_SmallAreaLowGrayLevelEmphasis +0.7814898×log-sigma-3-0-mm-3D_glszm_SmallAreaLowGrayLevelEmphasis+0.7188436×log-sigma-5-0-mm-3D_glcm_Imc2-0.7792080×square_glszm_SizeZoneNonUniformityNormalized +0.8779048×wavelet-LHL_firstorder_Median
